# Be Mindful of Your Metaphors about Microbes

**DOI:** 10.1128/mSphere.00431-21

**Published:** 2021-05-28

**Authors:** Jessica Maccaro

**Affiliations:** a Department of Entomology, University of California Riverside, Riverside, California, USA

**Keywords:** Darwinian medicine, antibiotic resistance, drug resistance evolution, ecology, martial metaphors, science communication

## Abstract

Metaphors are ubiquitous in science and have important implications for how we frame our research objectives as well as how we communicate to the public. This piece focuses on the power of metaphors to shape our attitude and actions toward antimicrobial-resistant bacteria. It begins by emphasizing the pervasiveness of war metaphors to describe bacteria. Then it highlights that, with this type of framing, the solutions follow a similar suit. Ultimately, this metaphorical framing can imply dangerously incorrect solutions to the problem of antibiotic resistance. I propose that we need metaphors that represent the problem of antimicrobial resistance as an ecological and evolutionary issue rather than a single bacterial enemy. I end by offering a new metaphor that does not downplay the healthy fear we should have for antimicrobial-resistant bacteria but acknowledges that living things evolve and self-preserve. This piece is a call to action to use metaphors that express microbes’ exceptional resilience rather than our brute strength in combat against them.

## COMMENTARY

Look at some of the phrases used to describe antibiotic-resistant bacteria in this 2009 review paper by Nerlich and James (1): “A new killer in our midst,” “powerful adversaries,” and “the battle against bacteria.”

Notice how they all describe resistant bacteria as an enemy to be feared and fought. As a result, the solutions to combat this “dangerous adversary” are framed as weapons: “Scientists show no mercy” with a “silver bullet” antibiotic designed to “give bacteria a sledgehammer blow” (1). Still, we might be “fighting a losing battle” and “running out of ammunition” (1). Such martial metaphors are ubiquitous in science and popular articles on antibiotic resistance.

But, why should it matter what metaphors we choose?

Metaphors are powerful, because they not only guide how to think about an idea but how to feel about an idea. This is important, because the way you feel about an idea often determines the action you take next. Let’s explore how these war metaphors can imply misleading solutions to the problem of antibiotic resistance and how we should seek to improve them.

First, by personifying antimicrobial-resistant bacteria as the enemy, it becomes easy to gloss over the fact that antibiotic resistance results from a bigger evolutionary process that will not disappear by killing any single enemy. Our metaphors around antibiotic resistance should more accurately reflect the evolutionary process so the actions we take are directed at the process rather than an individual “microbial foe.” Furthermore, presenting bacteria as adversaries can imply that they are strategic agents planning their next maneuver, when in reality they are just adapting to the current selective pressures (2). This language furthers the misconception that evolution has foresight if we think about bacterial evolution as a strategizing opponent. Moreover, the war metaphor is misleading because killing any number of the enemy seems like a victory in the context of war. So, any amount of antibiotic should weaken the enemy according to a war metaphor—but in reality, it can strengthen them. Without considering this subtlety, people might take antibiotics when they do not need them or not take the full round when they do. My father just the other day had a stomachache and popped a penicillin right away. This action made him feel as though he was getting the upper hand on the enemy. But instead, he might have selected for stronger pathogenic microbes and disturbed the ecological balance that kept them in check.

Which brings us to the second point: antibiotic resistance is an ecological problem—there is no bad microbe in a vacuum, but rather, they exist in a system of other microbes and their physical surroundings. Fortunately, the language around the human microbiome is becoming accurately ecological. For instance, a doctor would not describe irritable bowel syndrome as “a bad microbe” but rather as a “dysbiotic condition,” thus reflecting an ecological imbalance instead of a single enemy species. The term “dysbiosis” implies that the solution involves restoring a happy ecosystem. We need a metaphor that represents this mentality and encourages people to pursue a more ecological solution.

I will offer a starting point using the metaphor of the Hydra in Greek mythology. The Hydra is a snake-like creature with many heads. When anyone would attempt to cut off one of these heads, two would grow back in its place. The lone fighters that attempted to slay the Hydra slashed it over and over no matter how many more heads grew. Trying to destroy the Hydra with brute force only made it stronger. This is what can happen when we misuse antibiotics: in our efforts to “behead” disease, we provide an advantage to antibiotic-resistant microbes.

It was not until Hercules sought to understand the strange ways of the Hydra that he was able to slay it once and for all. He had to first chop off the heads and cauterize the fresh wounds immediately after. Because Hercules understood that slashing off each head as they cropped up would only make the problem worse, he targeted the process of exponential head regeneration rather than the heads themselves.

This metaphor highlights that we should guide our efforts toward the root of the problem. Although it still frames our relationship to antimicrobial-resistant bacteria as a fight, it directs our attention to the process that makes the Hydra dangerous. More specifically, this metaphor highlights that our aggressive interventions to destroy the Hydra (if indiscriminate and unthoughtful) actually drive the process that makes the Hydra even more dangerous. In the same sense, when we do not consider the evolutionary and ecological process that makes microbes so resilient, we escalate the very problem we are trying to eliminate—antibiotic-resistant bacteria. Addressing this problem is possible only when we deeply understand the nature of the microbes around us and the processes that underlie their success.

This metaphor does not diminish the healthy fear that we should have for antibiotic-resistant bacteria, but it suggests a more strategic path forward. While antibiotics are still often the best course of action, it is important to recognize the evolutionary process of resistance that they can escalate. Darwinian or evolutionary medicine is a huge step forward (3). This approach incorporates concepts from evolutionary biology to redefine and reframe disease and the questions and solutions surrounding it. Many have begun to embrace Darwinian medicine, because they acknowledge that living things evolve and self-preserve. Bacteria are masters of rapid evolution, so we should embrace metaphors that express their exceptional resilience rather than our brute strength in combat.
